# Influences of muscle mass loss and exercise habits and personality traits on lower limb motor function among university students

**DOI:** 10.1038/s41598-024-63089-6

**Published:** 2024-05-29

**Authors:** Nobuyuki Sano, Haruto Enoki, Reita Syutou, Ayumu Furukawa

**Affiliations:** 1 Department of Occupational Therapy, Faculty of Medical Science, Fukuoka International University of Health and Welfare, Fukuoka, Fukuoka Japan; 2 Fukuoka Rehabilitation Hospital, Fukuoka, Japan; 3 Seiai Rehabilitation Hospital, Onojo, Japan; 4 Karatsu Medical and Welfare Center for people with disabilities, Karatsu, Saga, Japan

**Keywords:** Health care, Medical research

## Abstract

Secondary sarcopenia, a risk factor even for young people, has attracted attention because of the deterioration of physical activity and nutritional status due to lifestyle change among university students. However, studies on the factors affecting motor function and their involvement are lacking. This cross-sectional study aimed to examine the influences of muscle mass loss and exercise and sleep habits on lower limb motor function, as well as the involvement of personality traits, in 101 university students. Approximately 6% of the participants had low skeletal muscle mass index, similar to previous reports, and that only exercise habits in high school were responsible for muscle mass loss (direct effect =  − 0.493; p < 0.05), wherease low skeletal muscle mass (direct effect =  − 0.539; p < 0.01) and current exercise habits (direct effect = 0.410; p < 0.01) were responsible for lower limb motor function. Additionaly, only the personality trait of high intellectual curiosity was involved in the establishment of exercise habits in high school, but no other personality traits showed a significant effect. In the prevention of secondary sarcopenia, encouraging sustained exercise habits while considering the influence of different personality traits is expected to prevent the decline in muscle mass and motor function.

## Introduction

Sarcopenia is a syndrome characterized by progressive and generalized loss of skeletal muscle mass. The European Working Group on Sarcopenia in Older People (EWGSOP) in 2010 recommends that sarcopenia be diagnosed by the presence of both low muscle mass and low muscle function (strength or performance)^1^. Sarcopenia is further classified into primary sarcopenia, which is an age-related decline in muscle quantity and quality, and secondary sarcopenia, which is related to specific diseases, activity level, and nutrition, regardless of age^1,2^. Secondary sarcopenia can also occur in young people^3^. Primary sarcopenia has been associated not only with major medical outcomes, including falls, fractures, hospitalization, long-term care, decreased activities of daily living (ADLs) and quality of life, and mortality in the elderly^4–6^, but also with health disadvantages, including decreased instrumental ADL performance^7^, drawing attention to the importance of sarcopenia in health care for the elderly. Contraily, for secondary sarcopenia unrelated to age, a review of data obtained from young people revealed that metabolic syndrome, physical inactivity, inadequate nutrition, inherent and perinatal factors, vitamin D deficiency, endocrinopathy, an imbalance of gut microbiota, neuromuscular diseases, organ failure, malignancy, and other inflammatory disorder were the etiologies in young people^3^. Some adverse effects caused by secondary sarcopenia, particularly in women, have been studied. For example, maternal prepregnancy underweight is associated with impairments in the intellectual domain (language comprehension index) of a child and an increased incidence of malnourishment among children^8^. Some of the major diseases in later life, such as coronary heart disease, hypertension, and type 2 diabetes in women, result from impaired intrauterine growth and development^9^. Furthermore, the body mass index (BMI) in youth correlates with the BMI in old age^10^, and while the effects of being underweight and sarcopenia are less obvious in youth, they are more likely to lead to develop decreased bone density and osteoporosis in old age and impaired glucose tolerance and diabetes^11,12^.

Recently, it has been estimated that more than one in every 10 young adults of most ethnicities has sarcopenia^3^. Additionally, the proportion of underweight and thin young adults in Japan is severely high, which is significantly higher than that of developed countries and comparable to that of developing countries^12^. In Kusakabe et al.’s study, approximately 5% of young people around 20 years of age had a markedly low skeletal muscle mass (SMI)^13^, which is defined as presarcopenia by EWGSOP in 2010^1^. Furthermore, in this study, the percentage of those with sarcopenia, which includes case with weak muscle strength due to decreased hand grip strength, was reduced to 1%, suggesting the possibility that young adults rarely suffer from muscle quality loss^13^. Given that the causes of secondary sarcopenia can be categorized as specific diseases, activity level, or nutrition^14^, inadequate nutrition and physical inactivity may be the primary causes among healthy adults. Regarding nutrition, young adults in their 20s may not be able to maintain sufficient muscle mass if they do not meet their daily protein intake^15^. Additionally, a study on women reported that distorted perceptions of their own body shape and the image of a desirable body shape lead to excessive dieting experiences and daily caloric restraint^16^. However, this trend is not limited to women in this genderless era, as men also have distorted perceptions of their body image and shape, which may negatively affect their physical activity and nutritional intake^17,18^.

Recently, other secondary sarcopenia exercise has been explored. Physical activity has many health benefits for young people; thus, in 2018, the World Health Organization (WHO) launched the new global action on physical activity, “More Active People for a Healthier World,” which set a new goal of a 15% relative reduction in the global prevalence of physical inactivity in youth and adults by 2030^19^. Globally, however, 81.0% of students aged 11–17 years were inadequately physically active in 2016 (77.6% for boys and 84.7% for girls)^20^. The prevalence of physical inactivity decreased significantly between 2001 and 2016 for boys (80.1% in 2001) but it did not change significantly for girls (85.1% in 2001)^20^. University students also tend to gain weight when they transition from secondary education to university because of considerable lifestyle changes that occur. This phenomenon is referred to as the “freshman 15” in the United States and stems from claims that the average weight gain during the first year of university is 15 pounds (6.8 kg)^21^. Graduating from high school is associated with a decrease in moderate-to-vigorous physical activity per day, and it decreases the dietary quality during high school and university graduation^22^. Althoug weight gain is an issue in North America, a common international challenge is the need to support the youth in post-secondary education in preventing physical inactivity and significant weight fluctuations.

Additionally, there have been several reports on the relationship between personality traits and individual characteristics affecting muscle mass. While BMI, an index of body size, has been used as a variable in these studies, some research has specifically examined the relationship between obesity and personality traits^23,24^. Although physical activity has positive effects on health, different personality traits tend to have different individual health-related perceptions and behaviors^24,25^. Particularly, many studies have investigated on personality traits based on the Big Five theory, one of the most popular international personality classifications, and in a review article, physical activity was positively associated with extraversion and conscientiousness, whereas neuroticism was negatively associated^26^. Openness to experience, particularly, may also result in stronger muscle strength due to an increased likelihood of engaging in physical activity, as openness favors novel and different types of activities^27,28^.

Therefore, the present study aimed to examine the effects of exercise and sleep habits on muscle mass loss in young adults and whether these factors also negatively affect lower limb motor function. Furthermore, we sought to clarify the characteristics of the effects of each personality trait on exercise and sleep habits and skeletal muscle mass loss.

## Methods

### Participants

University students aged ≥ 20 years were enrolled. This study was conducted from December 2021 to April 2022. As precautions, we explained to the participants that they should not eat or exercise for at least 1 h before the measurement, and those participants with pacemakers or other medical devices in their bodies could not be measured to ensure safety. Participants were recruited by explaining the study’s outline, presenting the schedule of multiple measurements before or after their university lectures, and obtaining their signatures using a research consent form during measurement. The exclusion criteria were those who had difficulty in holding a standing position or sitting up on their own due to a history of injury or disease. There is no definitive sample size calculation for structural equation modeling (SEM) analysis, whih serves as the primary analysis method in this study. As recommended by Boomsma, a sample size of ≥ 100 participants is advisable^29^. Therefore, following this previous study^12,15,30^, a sample size of 100 participants was assumed for recruitment in the present study.

### Instrumentation and outcomes

**Figure 1:**
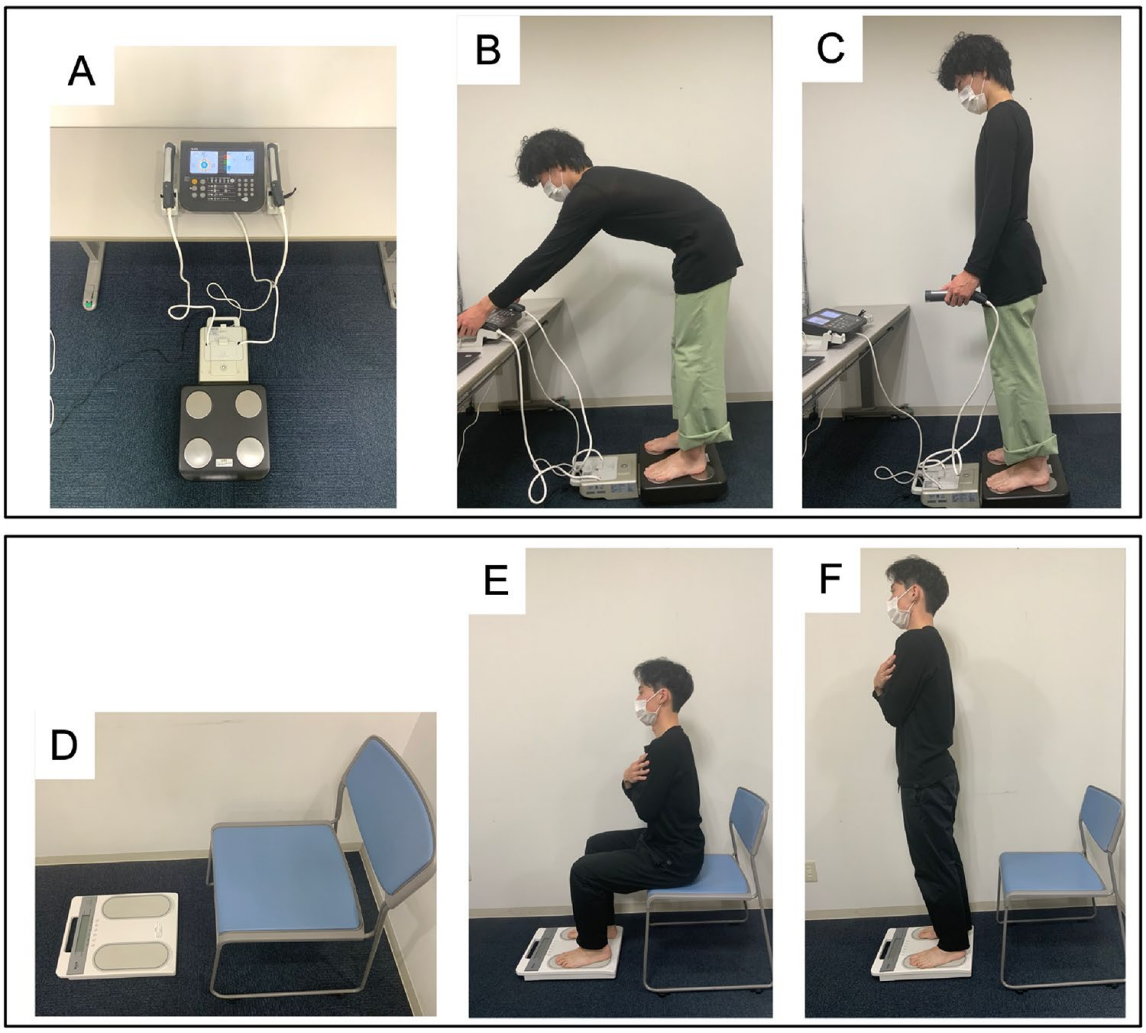
Body composition analyzer with an operating multi-frequency BIA and motor function analyzer. (**A**) Main body of the device (MC-780A-N, Tanita Co., Ltd., Tokyo). (**B**) Reach for and grasp the grip handle by oneself while on the measuring table. (**C**) Remain standing on the measuring table until the measurement is completed. (**D**) Main body of the device (Zaritz BM-220, Tanita Co., Ltd., Tokyo). (**E**) Image of the posture at the start of measurement. (**F**) Image during measurement holding standing position after standing up.

We used a body composition analyzer with an operating multifrequency bioelectrical impedance analyzer (BIA) (MC-780A-N, Tanita Co., Ltd., Tokyo) and a motor function analyzer (Zaritz BM-220, Tanita Co., Ltd., Tokyo) to measure and calculate the body composition and lower limb motor performance (Fig. 1). In both measurements, the participants were asked to stand barefoot on these devices, and the participant’s clothing was subtracted from their weight as 1.0 kg.

The following values were used in the evaluation using the body composition analyzer.

#### SMI

SMI is calculated as follows: appendicular skeletal muscle mass (ASM) (kg) divided by the square of height (m), which is an index of muscle mass for an individual’s body size. The equation for ASM measured by BIA used in this study has been published and demonstrated to be highly correlated with body composition measured using dual-energy X-ray absorptiometry^31^. Furthermore, referring to the Asian Working Group Sarcopenia (AWGS2014) criteria for SMI to determine sarcopenia^5^, male and female participants with SMI of < 7.0 and 5.7, respectively, were categorized as the significantly reduced skeletal muscle mass (Low SMI) group.

#### BMI

BMI, an index that expresses the balance between height and weight, is calculated as weight (kg)/height (m)/height (m). The standard range for BMI is from ≥ 18.5 to < 25 kg/m^2^^32^.

#### Total fat mass

Total fat mass is the amount of body fat estimated by using a body composition analyzer. BIA is a noninvasive examination technique used assess bone mass, fat mass, and fat-free mass. It involves passing weak currents with three different frequencies (5, 50, and 250 kHz) through the body using 8 electrodes: 2 for each sole and grip in a standing barefoot position. The difference in electric resistance is determined to derive the aforementioned mass measurements^31,33,34^.

#### Ratio of resistance to reactance (R/X)

Ratio of resistance to reactance using 50 kHz of BIA at both legs representing the balance between the intracellular and extracellular fluids. Resistance is strongly affected by extracellular fluid, whereas reactance is the resistance generated by the cell membrane and is also strongly affected by intracellular fluid. Therefore, the balance between the intracellular and extracellular fluids can be evaluated by looking at these ratios, with the lower the value, the more intracellular components there are, indicating that the intramuscular density is higher^34,35^.

For the motor function analyzer, participants sat in a sitting chair with a seat height of 40 cm from the floor and placed of their bare feet on the analyzer. In response to a cue given by the dedicated software, the participant quickly stood up from the chair with both hands crossed in front of the chest, stopped for 3 s, and then sat down in the chair again following the cue. Regarding the measurement method employed in this action, previous studies^36,37^ have reported intraclass correlation coefficients exceeding the excellence criterion, i.e., ≥ 0.7, with no variation in performances observed during our procedure review phase. Following confirmation that participants had sufficient understanding of the operation by reviewing the instructional video in the dedicated software and simulating the action, they performed the action only once. This enabled us to measure the participant’s maximum effort to stand up with his/her lower limbs, and to evaluate his/her lower limb muscle strength based on the load variation value (ground reaction force index) obtained at that time, elapsed time from standing up to stabilization, and balance ability based on the amount of displacement of the center of gravity on the left, right, and upper/lower sides^38,39^. The sampling frequency of the analyzer was 80 Hz, and data were acquired once every 0.0125 s.

The following values were used in the evaluation using the motor function analyzer.

#### Power (F/w)

This indicates the strength in the action of standing up, with a higher value indicating that the participants were able to stand up with greater strength in the lower limbs. It is the peak load point generated in standing up, defined as F, divided by the load at the stable point after standing up, defined as body weight (w), which means how many times the force of the body weight was applied to stand up. It is calculated as F/w (kgf/kg), which is the maximum load divided by the body weight^36,37^.

#### Speed (RFD/w)

This represents the quickness of standing up, with the higher the value, the quicker the participants could stand up. It is the maximum slope (rate of force development; RFD) of the graph between the point where the load begins to be applied and the point where the maximum load is recorded, calculated per kilogram of body weight. It is calculated as RFD/w (kgf/sec/kg), which is the value obtained by converting the amount of increase during a total of seven sections (0.0875 s), including one section where the maximum increase in load (ground reaction force) is recorded and three sections before and after that, into 1.0 s, and dividing by the body weight^36,37^.

#### Balance (Vx/Vw)

This indicates the degree of wobble from side to side in the action of standing up and the stable time (the time from the point when the maximum ground reaction force is recorded to the point when the wobble subsides), with higher values indicating more unstable standing movements. It is the value of variation in the left–right direction, defined as Vx (the value obtained by calculating the movement speed as an absolute value from the distance traveled to the left and right per section, and dividing by the stable time), divided by the load variation value defined as Vw (the value obtained by calculating the movement speed as an absolute value from the speed traveled up and down per section, and dividing by the stable time). It is calculated as Vx/Vw(mm/kg)^36^.

We used a web-based questionnaire and asked participants to complete the self-administered survey form.

#### Japanese version of the Ten Item Personality Inventory (TIPI-J)

TIPI-J is an evaluation scale that can determine an individual’s personality traits based on a combination of five factors. In response to 10 questions, the scale calculates the strength of extraversion (items 1 and 6), agreeableness (items 2 and 7), conscientiousness (items 3 and 8), neuroticism (items 4 and 9), and openness to experience (items 5 and 10) by self-rating the degree of agreement on a 7-point Likert scale ranging from 1 (completely disagree) to 7 (strongly agree). Items 2, 6, 8, 9, and 10 of TIPI-J were reverse-scored^40^.

#### Exercise and sleep habits

The participants were asked to respond to the number of times they exercised per week during their high school club activities (exercise_past), number of times they currently exercise per week (exercise_current), and average hours of sleep per week (sleep hours). Previous studies have reported that those who exercise regularly even once or twice a week are more effective in preventing the risk of developing serious diseases than those who do not exercise even once a week^41,42^. Therefore, with regard to past and current exercise habits, we converted the responses of those who answered that they exercised at least once per week into the following binary values: those who answered that they exercised at least once per week were considered to have a habit, and those who answered that they exercised 0 time per week were considered to have no habit.

### Procedures

Because personal information, such as body weight and body fat mass were important for university students, we ensured the anonymization of personal information from the data acquisition stage for the participants by implementing a system of research procedures. The participants were given a research ID by lottery and entered their research ID, age, sex, and height into a personal computer on which a dedicated software program linked to the body composition analyzer was installed. Then, the motion of standing up from a sitting position was measured using a motor function analyzer that can be continuously run using the same dedicated software as the body composition analyzer. The participants also completed a self-administered survey form using Google Forms with their own research IDs and completed the TIPI-J and the exercise and sleep habit survey. The study protocol was implemented in accordance with the ethical principles of the Declaration of Helsinki and was approved by the Ethics Review Committee of Fukuoka International University of Health and Welfare (approval number: 21-fiuhw-015). The participants were informed about this study and their written consent was obtained. Informed consent was obtained from all individuals in the images of Fig. 1 for publication in an online open access publication.

### Statistical analyses

After testing for normality, descriptive statistics were calculated for all participants and for each sex. Using the Welch test, we examined whether there were significant differences in body composition, motor function, personality traits, and sleep duration between the sex groups and between the groups of those who fell below the criteria for sarcopenia using SMI (low muscle mass group) and the others (normal group). The variables exercise_past and exercise_current were examined using either the chi-square or Fisher’s exact probability test. A correlation analysis (Pearson, polyserial, and polychoric) was used to calculate the correlation coefficient between each variable. The values of the correlation coefficients were presented as limited (0.00–0.25), fair (0.25–0.50), moderate (0.50–0.75), and excellent (0.75–1.0)^43^. To eliminate the possibility of multicollinearity in SEM analysis, we also checked for the relationships between variables with the correlation coefficients exceeding 0.9^44^.

**Figure 2:**
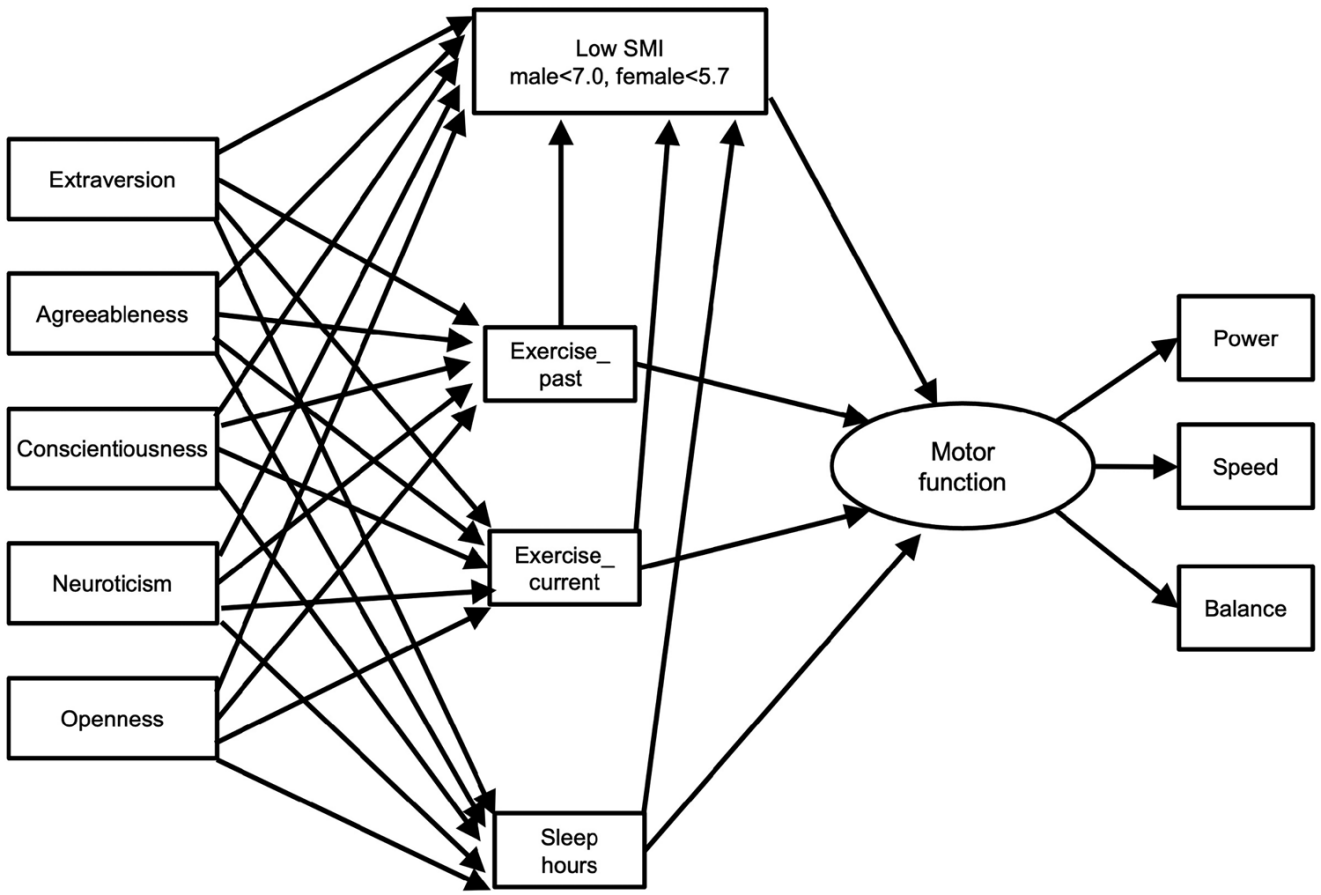
Hypothesized model. Oval represent latent variable (motor function) and rectangles represent observed variables (Low SMI, Exercise_past, Power, Extraversion, etc.)

Additionally, we used SEM to check the goodness of fit between the hypothesized model (Fig. 2) and the data collected in this study and to examine the structural relationships among the variables by obtaining the standardized path coefficients for the convergent analytical model. Our hypothesized model centered on a causal relationship with regular exercise (exercise_past and exercise_current) and sleep (sleep hours) habits as independent variables and Low SMI as the dependent variable, and predicted a negative effect of Low SMI and a positive effect of regular exercise and sleep habits on motor function (latent variable consisting of power, speed, and balance) in the null point. Furthermore, we predicted that the personality traits would act as a confounding factor for regular exercise and sleep habits and Low SMI, but that each personality factor would differ in its characteristics. In other words, conscientiousness was predicted to positively affect regular exercise and sleep habits, extraversion and openness were predicted to positively affect exercise habits, and neuroticism and agreeableness were predicted to negatively affect sleep habits, with the direct effects on Low SMI being partial or minimal^26–28^. The estimation method of SEM was performed using the modified weighted least squares method (WLSMV) with missing data, and we referred to the following fit indices: comparative fit index (CFI), Tucker Lewis Index (TLI), and root mean square error of approximation (RMSEA) with 90% confidence interval (CI)^45^. For CFI and TLI, a value of > 0.9 is the best model fit^46^. For RMSEA, a value of < 0.05 indicated a close fit, < 0.08 indicated a reasonable fit, and > 0.1 indicated a poor fit^47^. If the standardized path coefficient between the direct variables was significant and if the standardized path coefficient to the next latent variable was also significant, an indirect effect was also estimated by multiplying the two standardized path coefficients. Furthermore, we checked the goodness of fit of the SEM analysis for the hypothesized model for each sex. HAD17^48^ and Mplus version8^45^ were used in the analysis.

## Results

### Characteristics of the participants

The study participants were 101 university students (47 men and 54 women; mean age 20.59 ± 0.66 years, range: 20–24 years). The other descriptive statistics are shown in the Table 1. Two men and four women were categorized into the Low SMI group, representing 5.9% of all participants. In the normality test, the Kolmogorov–Smirnov test confirmed the normality for all variables, except for the agreeableness of personality traits and sleep hours. Additionally, descriptive statistics by sex are shown in the Table 2.

**Table 1:** Descriptive statistics.

	Mean	SD	Skewness	Kurtosis	Normality
Sex, n (%)	Male 47 (46.5), female 54 (53.5)				
Age, years	20.59 ± 0.66 (range: 20–24)				
SMI, kg/m^2^	7.206	1.082	0.387	−0.885	0.272
Low SMI, n (%)	Not applicable 95 (94.1), applicable 6 (5.9)				
BMI, kg/m^2^	21.041	2.278	0.531	0.492	0.665
Total fat mass, kg	13.353	3.819	0.019	− 0.195	0.985
R/X, Ω	10.076	1.434	0.357	− 0.644	0.559
Power, kgf/kg	1.452	0.141	0.272	− 0.654	0.653
Speed, kgf/s/kg	10.081	2.282	− 0.126	1.043	0.819
Balance, mm/kg	1.021	0.433	0.966	0.46	0.081
Extraversion, point	4.094	1.399	0.074	− 0.682	0.067
Agreeableness, point	5.49	0.857	− 0.406	− 0.421	0.022
Conscientiousness, point	3.644	1.258	0.072	− 0.642	0.28
Neuroticism, point	4.02	1.206	− 0.117	− 0.111	0.195
Openness, point	4.089	1.028	0.178	− 0.656	0.154
Exercise_past, n (%)	Had 89 (88.1), no 12 (11.9)				
Exercise_current, n (%)	Have 62 (61.4), no 39 (38.6)				
Sleep hours, hour	6.109	1.038	0.27	1.115	0.001

*SD* standard deviation.

**Table 2:** Descriptive statistics by sex.

	Male (n = 47)			Female (n = 54)			Welch test
	Mean	SD	SE	Mean	SD	SE	P value
SMI, kg/m^2^	8.128	0.717	0.105	6.404	0.596	0.081	0.000
Low SMI, n (%)	Not applicable 45(95.7%), applicable 2(4.3%)			Not applicable 50(92.6%), applicable 4(7.4%)			χ^2^ = 0.447 p = 0.504 Fisher’s = 0.683
BMI, kg/m^2^	21.872	2.561	0.373	20.317	1.718	0.234	0.001
Total Fat mass, kg	12.117	4.289	0.626	14.43	3.002	0.409	0.003
R/X, Ω	9.074	0.930	0.136	10.948	1.211	0.165	0.000
Power, kgf/kg	1.549	0.117	0.017	1.367	0.101	0.014	0.000
Speed, kgf/sec/kg	11.083	2.228	0.325	9.209	1.963	0.267	0.000
Balance, mm/kg	0.773	0.251	0.037	1.238	0.443	0.060	0.000
Extraversion, point	4.106	1.482	0.216	4.083	1.338	0.182	0.935
Agreeableness, point	5.457	0.89	0.130	5.519	0.835	0.114	0.724
Conscientiousness, point	3.67	1.244	0.181	3.62	1.281	0.174	0.843
Neuroticism, point	4.064	1.271	0.185	3.981	1.157	0.157	0.736
Openness, point	4.351	1.063	0.155	3.861	0.949	0.129	0.017
Exercise_past, n (%)	Had 43(91.5%), no 4(8.5%)			Had 46(85.2%), no 8(14.8%)			χ2 = 0.954 p = 0.329 Fisher’s = 0.373
Exercise_current, n (%)	Have 37(78.7%), no 10(21.3%)			Have 25(46.3%), no 29(53.7%)			χ2 = 11.147 p = 0.001 Fisher’s = 0.001
Sleep hours, hour	6.149	1.161	0.169	6.074	0.929	0.126	0.724

*SD* standard deviation, *SE* standard error.

### Comparison between the male and female groups

Comparing the male and female groups (Table 2), SMI (p < 0.01), BMI (p < 0.01) power (p < 0.01), speed (p < 0.01), openness (p = 0.017), and exercise_current (p < 0.01)) tended to be significantly higher in the male group, wherease total fat mass (p < 0.01), R/X (p < 0.01), and balance (p < 0.01) tended to be significantly lower in the male group.

### Comparison between the normal and low SMI groups

Comparing the Low SMI group with the normal group (Table 3), BMI (p < 0.01), balance (p = 0.027), and openness tended to be significantly higher in the normal group (p = 0.046). Additionally, the normal group was more likely to be habitual in exercise_past (p = 0.021). The R/X value tended to be suggestively higher (p = 0.074) and the power tended to be suggestively lower (p = 0.081) in the Low SMI group.

**Table 3:** Comparison between normal group and low SMI group.

	Normal group (n = 95)			Group with Low SMI (n = 6)			Welch test
	Mean	SD	SE	Mean	SD	SE	P value
SMI, kg/m^2^	7.283	1.059	0.109	5.983	0.677	0.276	0.004
BMI, kg/m^2^	21.215	2.210	0.227	18.283	1.496	0.611	0.003
Total Fat mass, kg	13.442	3.845	0.394	11.950	3.344	1.365	0.335
R/X, Ω	10.007	1.422	0.146	11.167	1.260	0.514	0.074
Power, kgf/kg	1.458	0.142	0.015	1.362	0.107	0.044	0.081
Speed, kgf/sec/kg	10.184	2.243	0.230	8.459	2.501	1.021	0.155
Balance, mm/kg	0.987	0.410	0.042	1.563	0.460	0.188	0.027
Extraversion, point	4.137	1.392	0.143	3.417	1.463	0.597	0.289
Agreeableness, point	5.489	0.866	0.089	5.500	0.775	0.316	0.976
Conscientiousness, point	3.663	1.264	0.130	3.333	1.211	0.494	0.544
Neuroticism, point	4.032	1.213	0.124	3.833	1.169	0.477	0.702
Openness, point	4.132	1.035	0.106	3.417	0.665	0.271	0.046
Exercise_past, n (%)	Had 86 (85.1%), no 9 (8.9%)			Had 3 (3.0%), no 3 (3.0%)			χ^2^ = 8.853 p = 0.003 Fisher’s = 0.021
Exercise_current, n (%)	Had 58 (57.4%), no 37 (36.6%)			Had 4 (4.0%), no 2 (2.0%)			χ^2^ = 0.075 p = 0.784 Fisher’s = 1.000
Sleep hours, hour	6.105	1.057	0.108	6.167	0.753	0.307	0.856

*SD* standard deviation, *SE* standard error.

### Relationship between the variables

The correlation analysis revealed no relationship between variables with high correlation coefficients (Table 4). The results of the polychoric or polyserial correlation analysis showed that the variable Low SMI had significantly moderate or fair correlation coefficients with BMI (r =  − 0.723, p < 0.01), exercise_past (r =  − 0.596, p < 0.01), and balance (r = 0.488, p < 0.01) and suggestively fair correlation coefficients with openness (r =  − 0.340, p = 0.077), power (r =  − 0.364, p = 0.078), and speed (r =  − 0.346, p = 0.066).

**Table 4:** Correlation analysis between variables.

	SMI	BMI	Fat	R/X	Power	Speed	Balance	Extra	Agree	Cons	Neuro	Open	Past	Current	Sleep
SMI	1.000														
BMI	− 0.723**	1.000													
Fat	** − 0.197**	0.584**	1.000												
R/X	**0.380**	− 0.524**	0.149	1.000											
Power	** − 0.364 + **	0.259**	− 0.128	− 0.386**	1.000										
Speed	** − 0.346 + **	0.286**	− 0.031	− 0.315**	0.713**	1.000									
Balance	0.488**	− 0.419**	− 0.009	0.430**	− 0.606**	− 0.537**	1.000								
Extra	* − 0.247*	**0.125**	**0.101**	** − 0.081**	**0.062**	**0.044**	** − 0.061**	1.000							
Agree	* − 0.005*	** − 0.012**	** − 0.007**	**0.048**	**0.075**	**0.042**	**0.003**	* − 0.070*	1.000						
Cons	* − 0.122*	**0.059**	**0.048**	** − 0.087**	** − 0.012**	** − 0.068**	**0.084**	0.227*	*0.069*	1.000					
Neuro	* − 0.077*	**0.088**	**0.143**	** − 0.070**	** − 0.065**	** − 0.057**	** − 0.023**	− 0.194*	− 0.358**	* − 0.126*	1.000				
Open	* − 0.340* +	0.342**	**0.142**	− 0.269**	0.236**	**0.124**	** − 0.186 + **	0.325**	*0.143*	*0.188* +	* − 0.051*	1.000			
Past	− 0.596**	0.519**	**0.063**	− 0.420**	**0.039**	** − 0.013**	** − 0.229**	0.327*	* − 0.291* +	*0.054*	* − 0.083*	0.353*	1.000		
Current	*0.071*	0.416**	** − 0.021**	− 0.542**	0.253*	0.256*	** − 0.194**	*0.193*	*0.011*	0.289*	*0.173*	*0.146*	*0.173*	1.000	
Sleep	*0.036*	0.080	**0.065**	** − 0.136**	** − 0.096**	**0.024**	**0.059**	*0.081*	* − 0.005*	0.237*	*0.012*	*0.099*	*0.244*	0.340**	1.000

Bold were analyzed by polyserial (continuous variables × ordinal variables), italics by polychoric (ordinal variables × ordinal variables), and others by Pearson correlation analysis.

*SMI* low SMI, *FAT* total fat mass, *Extra* extraversion, *Agree* agreeableness, *Cons* conscientiousness, *Neuro* neuroticism, *Open* openness, *Past* exercise_past, *Current* exercise_current, *Sleep* sleep hours.

### SEM of the hypothesized model

The results of the SEM analysis of the hypothesized model created in this study showed that the fit indices were CFI of 0.935, TLI of 0.860, and RMSEA of 0.048 (90% CI: 0.000–0.097), CFI was the best model fit and RMSEA was a close fit criterion. However, the standardized path coefficient from low SMI to motor function was − 1.259 (p < 0.05). Given that the standardized path coefficient ranged between − 1 and 1, this standardized path coefficient was considered a result indicating an unsuitable solution (Supplementary information file 2).

Therefore, a modified model was created by removing the paths of exercise_past and sleep hours that were not significant among the paths for the motor function as objective variables and was analyzed again with SEM. As a result, the fit indices were CFI of 0.921, TLI of 0.842, and RMSEA of 0.051 (90% CI: 0.000–0.097). CFI was the best model fit, RMSEA was an almost close fit criterion, and no path coefficient indicating an inappropriate solution was found (Table 5). There was a significant effect of Low SMI (direct effect =  − 0.539; p < 0.01) and exercise_current (direct effect = 0.410; p < 0.01) on motor function. Only exercise_past (direct effect =  − 0.493; p < 0.05) had a significant effect on Low SMI. Regarding the personality traits, a significant effect was observed on openness on exercise_past (direct effect = 0.387; p < 0.05). There was a suggestive effect from agreeableness on exercise_past (direct effect =  − 0.451; p = 0.075), and from conscientiousness on exercise_current (direct effect = 0.228; p = 0.058) and sleep hours. No personality traits had a direct effect on Low SMI (Table 5). The indirect effect of exercise_past on motor function via Low SMI (indirect effect = 0.266; p = 0.058) was at a suggestive level. The indirect effect from openness to Low SMI via exercise_ past (indirect effect =  − 0.191 (p = 0.107)) was not significant. We conducted SEM analysis on separate models for males and females. However, we found that the goodness of fit of the original hypothesis was poor in both cases (Supplementary information files 3 and 4). Specifically, the fit indices of the hypothesized model with male participants were as follows: CFI of 0.789; TLI of 0.545; RMSEA of 0.083 (90% CI: 0.000–0.151). The fit indices of the hypothesized model with female participants were as follows: CFI of 0.665; TLI of 0.279; RMSEA of 0.083 (90% CI: 0.000–0.145).

**Table 5:** Standardized path coefficient of SEM analysis of the modified model.

Modified model				
Two-tailed	Estimate	SE	Estimate/SE	P-value
Motor function	By			
Power	0.840	0.053	15.953	0.000
Speed	0.760	0.051	15.016	0.000
Balance	− 0.772	0.071	− 10.884	0.000
Motor function	On			
Low SMI	− 0.539	0.118	− 4.553	0.000
Exercise_current	0.410	0.148	2.765	0.006
Low SMI	On			
Extraversion	0.138	0.243	0.568	0.570
Agreeableness	− 0.199	0.258	− 0.772	0.440
Conscientiousness	0.174	0.165	1.050	0.294
Neuroticism	0.000	0.193	− 0.002	0.999
Openness	− 0.242	0.251	− 0.965	0.335
Exercise_past	− 0.493	0.226	− 2.185	0.029
Exercise_current	0.170	0.135	1.255	0.209
Sleep hours	0.099	0.099	1.001	0.317
Exercise_past	On			
Extraversion	0.165	0.196	0.845	0.398
Agreeableness	− 0.451	0.254	− 1.779	0.075
Conscientiousness	− 0.052	0.182	− 0.286	0.775
Neuroticism	− 0.184	0.162	− 1.141	0.254
Openness	0.387	0.165	2.344	0.019
Exercise_current	On			
Extraversion	0.168	0.128	1.311	0.190
Agreeableness	0.078	0.142	0.551	0.582
Conscientiousness	0.228	0.120	1.894	0.058
Neuroticism	0.219	0.141	1.553	0.120
Openness	0.041	0.139	0.291	0.771
Sleep hours	On			
Extraversion	0.026	0.118	0.224	0.823
Agreeableness	− 0.034	0.121	− 0.281	0.779
Conscientiousness	0.210	0.109	1.926	0.054
Neuroticism	0.003	0.110	0.024	0.981
Openness	0.032	0.124	0.262	0.794
R-SQUARE				
Exercise_past	0.380	0.205	1.857	0.063
Exercise_current	0.125	0.084	1.492	0.136
Sleep hours	0.051	0.046	1.118	0.264
Low SMI	0.390	0.150	2.596	0.009
Power	0.705	0.088	7.976	0.000
Speed	0.578	0.077	7.508	0.000
Balance	0.596	0.110	5.442	0.000
Motor function	0.375	0.147	2.557	0.011

Fit indices: CFI = 0.921, TLI = 0.842, RMSEA = 0.051, 90% IC [0.000, 0.097].

## Discussion

The present study tested a hypothesized model in which significant muscle mass loss affects the lower limb motor function, and exercise and sleep habits and personality traits also affect significant muscle mass loss, in university students aged ≥ 20 years. The results of the examination of the hypothetical model by performing a SEM analysis showed that the modified model in which the paths between variables that were non-significant were removed from the hypothetical model generated in this study showed little change when compared with the original hypothesis, and both models had a level of fit that could be adopted as a criterion for close fit. Therefore, it can be concluded that the modified model was finally adopted in the analysis using the data obtained in this study.

The structural relationships in the modified model showed that only exercise habits in high school had a significant effect on significant muscle mass loss. University students who were previously habituated to exercise through participation in athletic clubs during high school have been reported to exhibit higher mean SMI values^49^. These findings underscore the importance of physical activity starting from a young age, as emphasized by WHO^19^, which may have implications not only for the prevention of obesity and hypertension^50–52^ but also for averting secondary sarcopenia. Additionally, muscle mass loss and lack of current exercise habits had a significant effect on lower limb motor function, given that the detrimental effect of muscle mass loss on motor function may outweigh the impact of lacking current exercise habits. Previous studies examining age-related changes in muscle mass among Japanese individuals have highlighted the importance of lower limb muscle mass. Although upper limb muscles show minimal age-related changes, the decline in lower limbs among Japanese people begins in their 20s, with the most substantial age-related decline in observed in the lower limb muscle mass compared with all other body parts^53,54^. Consequently, even younger individuals who do not currently experience major life-related issues may faces challenges in maintaining mobility as they age, especially due to age-related declines in lower limb muscle mass, despite having exercise habits in the past^49^. No paths were found for the personality traits to directly influence muscle mass loss, and the only significant relationship was the positive influence of openness to experience on exercise habits in high school. The other findings assume that a high level of conscientiousness tends to form the current exercise and sleep habits more, whereas a high level of agreeableness may make it difficult to form exercise habits in high school. This result supports the relationship reported in previous studies^26^, suggesting that conscientiousness may be important in developing adherence behavior in people with a high sense of purpose and self-control, whereas agreeabeleness may result from the fact that individuals are easily influenced by the temptations and enticements found in their surroundings, making it difficult to establish their own regular behavioral patterns. These findings suggest that personality traits have partial effects on the past and current exercise habits, but do not have a strong causal effect on the body components such as muscle mass and fat, or even motor performance. In other words, the lack of regular exercise habits in high school may lead to the current marked decline of muscle mass. Furthermore, the marked decline of muscle mass and the lack of current exercise habits are predicted to cause a decline in motor function of the lower limbs when standing up. Additionally, it was inferred that those with intellectually curious personalities are more likely to form exercise habits through club activities in high school, and previous studies supported the tendency for high openness to experiementation, preferring various activities and engaging in physical activity^27,28^.

In the present study population, approximately 6% of healthy adults in their 20s met the criteria for presarcopenia, confirming a risk associated with a decline in lower limb motor function. Approximately 90% of the participants had a habit of exercising in high school, indicating that the majority had engaged in regular exercise in the past. However, approximately 60% of the participants reported having a current exercise habit, suggesting that approximately 30% may have reduced their exercise habits. This trend aligns with reports indicating that students are more likely to experience a decline in physical activity during their university years than during high school^22^. Regarding sleep duration, in international reports, Japanese people are recognized for sleeping shorter hours, as evidenced by an organization for Economic Co-operation and Development survey showing that the average sleep duration of Japanese individuals was approximately 440 min, the shortest period among the 33 countries surveyed^55^. Additionally, a survey conducted by Japan's Ministry of Health, Labour and Welfare on sleep hours revealed that approximately 70–80% of Japanese individuals in their 20 s sleep for < 7 h^56^. Although a previous study^57^ involving Japanese university students reported an average sleep duration of < 7 h, participants in the present study tended to sleep for fewer hours than those reported previously.

The comparison between the Low SMI and normal groups showed significant difference in terms of lower BMI, balance of motor function, openness personality traits, and absence of an exercise habit in high school, suggesting a trend toward higher R/X values and lower power of motor function. BMI and SMI differ only in that they use total body weight or ASM, respectively, in their calculation formulas, resulting in similar trends in the BMI values. However, BMI and SMI have been used in predicting musculoskeletal injuries^58^ and determining the prognosis of patients with cancer^59,60^. A higher R/X value indicates a smaller percentage of intracellular muscle fibers^34^. Therefore, muscles density was lower in the group with Low SMI. In a study concerning muscle density and motor function, Goodpaster et al.^61^ suggested that a significant reduction in leg strength may be attributed to muscle quality, which is influenced by intramuscular fat infiltration and myofibrosis^62^. This notion is supported by the correlation shown in Table 3, indicating a fair correlation coefficient between R/X and the three motor function variables (power, speed, and balance). Besides, in a recently reported survey of Japanese university students, 7.4% were found to have presarcopenia^13^, a similar to the percentage in the present study. Factors associated with sarcopenia were physical activity in male and energy intake in female. Although the nutritional status was not assessed in this study, the study by Kusakabe et al. suggests that there may be different factors related to muscle mass and strength in male and female, suggesting the need for further research^13^.

There are several limitations to this study. First, because this is a cross-sectional study, the results obtained from the analysis in this study do not indicate a clear causal relationship. However, using the SEM approach, we could find the structural relationships, and future studies should examine the causal relationships based on this hypothesis. Second, the data on exercise and sleep habits in the questionnaire were not objective, but instead they were retrospective data, which raises doubts about the accuracy of the information. In addition, we did not assess the intensity of exercise or the total exercise time in a week, which have been used in previous studies^13,22^, and asked only about the number of exercise sessions in a week. In future research, exercise and sleep habits can be easily obtained using activity tracker^63^ and so on; thus, it is necessary to conduct research that compares the data with information obtained using such objective devices. Finally, although we excluded the individuals with a history of disease or injury that made it difficult for them to stand or sit up during the inclusion phase, we cannot exclude the possibility that other disease-related secondary sarcopenia was included. Additionally, the participants’ body water content was not strictly controlled by diet and exercise, which may have distorted the body composition values due to the individual measurement errors.

### Supplementary Information

Supplementary Information 1.

Supplementary Information 2.

## Data Availability

Data is provided within supplementary information files 1.
