# Supplementary material for: Influences of muscle mass loss and exercise habits and personality traits on lower limb motor function among university students
Source: Sci Rep. 2024 May 29;14:12341. doi: 10.1038/s41598-024-63089-6 (PMC11136959; doi:10.1038/s41598-024-63089-6)
Supplement: Supplementary file 2 — Supplementary Information 2. [file 41598_2024_63089_MOESM2_ESM.docx]

**Influences of muscle mass loss and exercise habits and personality traits on lower limb motor function among university students**

**Nobuyuki Sano*, Haruto Enoki, Reita Syutou, Ayumu Furukawa**

***Corresponding author: Nobuyuki Sano**

**E-mail: sanokichi09094@gmail.com. (NS)**

**Supplementary Information file 2. Standardized path coefficient of SEM analysis of the hypothesized model**

| **Original model** | |  |  |  |  |
| --- | --- | --- | --- | --- | --- |
| Two-Tailed | | Estimate | SE | Estimate/SE | P-Value |
| Motor function | | By |  |  |  |
|  | Power | 0.829 | 0.053 | 15.570 | 0.000 |
|  | Speed | 0.784 | 0.049 | 15.856 | 0.000 |
|  | Balance | −0.754 | 0.072 | −10.462 | 0.000 |
| Motor function | | On |  |  |  |
|  | Low SMI | **−1.259** | 0.605 | −2.082 | 0.037 |
|  | Exercise_past | −0.861 | 0.606 | −1.421 | 0.155 |
|  | Exercise_current | 0.635 | 0.287 | 2.214 | 0.027 |
|  | Sleep hours | 0.100 | 0.140 | 0.713 | 0.476 |
| Low SMI | | On |  |  |  |
|  | Extraversion | 0.062 | 0.113 | 0.547 | 0.585 |
|  | Agreeableness | −0.012 | 0.104 | −0.118 | 0.906 |
|  | Conscientiousness | 0.133 | 0.095 | 1.396 | 0.163 |
|  | Neuroticism | 0.063 | 0.098 | 0.635 | 0.526 |
|  | Openness | −0.165 | 0.134 | −1.228 | 0.219 |
|  | Exercise_past | −0.738 | 0.166 | −4.442 | 0.000 |
|  | Exercise_current | 0.224 | 0.136 | 1.653 | 0.098 |
|  | Sleep hours | 0.112 | 0.093 | 1.204 | 0.228 |
| Exercise_past | | On |  |  |  |
|  | Extraversion | 0.207 | 0.195 | 1.062 | 0.288 |
|  | Agreeableness | −0.290 | 0.265 | −1.095 | 0.273 |
|  | Conscientiousness | 0.037 | 0.185 | 0.201 | 0.841 |
|  | Neuroticism | −0.087 | 0.179 | −0.486 | 0.627 |
|  | Openness | 0.383 | 0.164 | 2.340 | 0.019 |
| Exercise_current | | On |  |  |  |
|  | Extraversion | 0.185 | 0.128 | 1.444 | 0.149 |
|  | Agreeableness | 0.062 | 0.144 | 0.430 | 0.667 |
|  | Conscientiousness | 0.251 | 0.117 | 2.145 | 0.032 |
|  | Neuroticism | 0.229 | 0.138 | 1.651 | 0.099 |
|  | Openness | 0.039 | 0.139 | 0.278 | 0.781 |
| Sleep hours | | On |  |  |  |
|  | Extraversion | 0.025 | 0.118 | 0.215 | 0.830 |
|  | Agreeableness | −0.037 | 0.121 | −0.307 | 0.759 |
|  | Conscientiousness | 0.207 | 0.109 | 1.900 | 0.057 |
|  | Neuroticism | −0.002 | 0.110 | −0.017 | 0.986 |
|  | Openness | 0.033 | 0.124 | 0.269 | 0.788 |
| R-SQUARE | |  |  |  |  |
|  | Exercise_past | 0.318 | 0.180 | 1.769 | 0.077 |
|  | Exercise_current | 0.146 | 0.087 | 1.674 | 0.094 |
|  | Sleep hours | 0.050 | 0.045 | 1.102 | 0.270 |
|  | Low SMI | 0.674 | 0.168 | 4.018 | 0.000 |
|  | Power | 0.688 | 0.088 | 7.785 | 0.000 |
|  | Speed | 0.614 | 0.077 | 7.928 | 0.000 |
|  | Balance | 0.569 | 0.109 | 5.231 | 0.000 |
|  | Motor function | 0.690 | 0.333 | 2.070 | 0.038 |

Fit indices: CFI = 0.935, TLI = 0.860, RMSEA = 0.048, 90％ IC [0.000, 0.097]

The underlined paths from Low SMI to motor function were those considered unsuitable because their standardized path coefficients exceed 1.00.

**Influences of muscle mass loss and exercise habits and personality traits on lower limb motor function among university students**

**Nobuyuki Sano*, Haruto Enoki, Reita Syutou, Ayumu Furukawa**

***Corresponding author: Nobuyuki Sano**

**E-mail: sanokichi09094@gmail.com. (NS)**

**Supplementary Information file 3. Standardized path coefficient of SEM analysis of the hypothesized model with male participant**

| **Original model** | |  |  |  |  |
| --- | --- | --- | --- | --- | --- |
| Two-Tailed | | Estimate | SE | Estimate/SE | P-Value |
| Motor function | | By |  |  |  |
|  | Power | 0.590 | 0.108 | 5.489 | 0.000 |
|  | Speed | 0.690 | 0.105 | 6.593 | 0.000 |
|  | Balance | -0.707 | 0.104 | -6.774 | 0.000 |
| Motor function | | On |  |  |  |
|  | Low SMI | **-8.247** | 24.633 | -0.335 | 0.738 |
|  | Exercise_past | **-7.504** | 23.959 | -0.313 | 0.754 |
|  | Exercise_current | -0.817 | 6.207 | -0.132 | 0.895 |
|  | Sleep hours | **1.211** | 3.337 | 0.363 | 0.717 |
| Low SMI | | On |  |  |  |
|  | Extraversion | 0.053 | 0.163 | 0.324 | 0.746 |
|  | Agreeableness | 0.017 | 0.073 | 0.236 | 0.813 |
|  | Conscientiousness | 0.067 | 0.199 | 0.335 | 0.738 |
|  | Neuroticism | 0.026 | 0.087 | 0.304 | 0.761 |
|  | Openness | -0.044 | 0.136 | -0.327 | 0.744 |
|  | Exercise_past | -0.951 | 0.112 | -8.493 | 0.000 |
|  | Exercise_current | -0.185 | 0.409 | -0.453 | 0.650 |
|  | Sleep hours | 0.157 | 0.213 | 0.737 | 0.461 |
| Exercise_past | | On |  |  |  |
|  | Extraversion | 0.756 | 0.500 | 1.512 | 0.131 |
|  | Agreeableness | -0.199 | 1.073 | -0.186 | 0.853 |
|  | Conscientiousness | -0.165 | 0.506 | -0.327 | 0.744 |
|  | Neuroticism | 0.134 | 0.581 | 0.231 | 0.818 |
|  | Openness | -0.213 | 0.482 | -0.442 | 0.658 |
| Exercise_current | | On |  |  |  |
|  | Extraversion | 0.264 | 0.240 | 1.102 | 0.270 |
|  | Agreeableness | 0.007 | 0.221 | 0.030 | 0.976 |
|  | Conscientiousness | 0.647 | 0.155 | 4.170 | 0.000 |
|  | Neuroticism | -0.023 | 0.187 | -0.124 | 0.901 |
|  | Openness | -0.228 | 0.341 | -0.668 | 0.504 |
| Sleep hours | | On |  |  |  |
|  | Extraversion | -0.157 | 0.222 | -0.705 | 0.481 |
|  | Agreeableness | 0.072 | 0.208 | 0.347 | 0.728 |
|  | Conscientiousness | 0.118 | 0.215 | 0.546 | 0.585 |
|  | Neuroticism | -0.035 | 0.179 | -0.198 | 0.843 |
|  | Openness | 0.194 | 0.312 | 0.623 | 0.533 |
| R-SQUARE | |  |  |  |  |
|  | Exercise_past | 0.522 | 0.517 | 1.011 | 0.312 |
|  | Exercise_current | 0.445 | 0.221 | 2.018 | 0.044 |
|  | Sleep hours | 0.052 | 0.072 | 0.724 | 0.469 |
|  | Low SMI | 0.958 | 0.147 | 6.521 | 0.000 |
|  | Power | 0.348 | 0.127 | 2.745 | 0.006 |
|  | Speed | 0.476 | 0.144 | 3.297 | 0.001 |
|  | Balance | 0.500 | 0.148 | 3.387 | 0.001 |
|  | Motor function | Undefined |  |  |  |

Fit indices: CFI = 0.789, TLI = 0.545, RMSEA = 0.083, 90％ IC [0.000, 0.151]

The underlined paths from Low SMI, Exercise_past, and Sleep hours to motor function were those considered unsuitable because their standardized path coefficients exceed 1.00.

**Influences of muscle mass loss and exercise habits and personality traits on lower limb motor function among university students**

**Nobuyuki Sano*, Haruto Enoki, Reita Syutou, Ayumu Furukawa**

***Corresponding author: Nobuyuki Sano**

**E-mail: sanokichi09094@gmail.com. (NS)**

**Supplementary Information file 4. Standardized path coefficient of SEM analysis of the hypothesized model with female participant**

| **Original model** | |  |  |  |  |
| --- | --- | --- | --- | --- | --- |
| Two-Tailed | | Estimate | SE | Estimate/SE | P-Value |
| Motor function | | By |  |  |  |
|  | Power | 0.822 | 0.113 | 7.252 | 0.000 |
|  | Speed | 0.641 | 0.103 | 6.235 | 0.000 |
|  | Balance | -0.756 | 0.121 | -6.228 | 0.000 |
| Motor function | | On |  |  |  |
|  | Low SMI | **-1.221** | 0.975 | -1.251 | 0.211 |
|  | Exercise_past | -0.802 | 0.790 | -1.015 | 0.310 |
|  | Exercise_current | 0.398 | 0.708 | 0.562 | 0.574 |
|  | Sleep hours | 0.340 | 0.382 | 0.888 | 0.374 |
| Low SMI | | On |  |  |  |
|  | Extraversion | -0.100 | 0.183 | -0.546 | 0.585 |
|  | Agreeableness | -0.209 | 0.230 | -0.908 | 0.364 |
|  | Conscientiousness | 0.045 | 0.157 | 0.288 | 0.774 |
|  | Neuroticism | -0.107 | 0.176 | -0.608 | 0.543 |
|  | Openness | -0.030 | 0.162 | -0.187 | 0.851 |
|  | Exercise_past | -0.613 | 0.259 | -2.368 | 0.018 |
|  | Exercise_current | 0.408 | 0.340 | 1.202 | 0.229 |
|  | Sleep hours | 0.293 | 0.169 | 1.728 | 0.084 |
| Exercise_past | | On |  |  |  |
|  | Extraversion | 0.112 | 0.357 | 0.314 | 0.754 |
|  | Agreeableness | -0.543 | 0.279 | -1.951 | 0.051 |
|  | Conscientiousness | -0.086 | 0.311 | -0.275 | 0.783 |
|  | Neuroticism | -0.264 | 0.220 | -1.200 | 0.230 |
|  | Openness | 0.623 | 0.195 | 3.197 | 0.001 |
| Exercise_current | | On |  |  |  |
|  | Extraversion | 0.360 | 0.182 | 1.980 | 0.048 |
|  | Agreeableness | 0.098 | 0.172 | 0.569 | 0.569 |
|  | Conscientiousness | 0.074 | 0.185 | 0.401 | 0.688 |
|  | Neuroticism | 0.453 | 0.175 | 2.590 | 0.010 |
|  | Openness | 0.004 | 0.182 | 0.021 | 0.984 |
| Sleep hours | | On |  |  |  |
|  | Extraversion | 0.131 | 0.134 | 0.974 | 0.330 |
|  | Agreeableness | -0.137 | 0.188 | -0.730 | 0.465 |
|  | Conscientiousness | 0.259 | 0.146 | 1.772 | 0.076 |
|  | Neuroticism | 0.054 | 0.182 | 0.297 | 0.766 |
|  | Openness | 0.004 | 0.161 | 0.022 | 0.982 |
| R-SQUARE | |  |  |  |  |
|  | Exercise_past | 0.429 | 0.181 | 2.378 | 0.017 |
|  | Exercise_current | 0.264 | 0.167 | 1.576 | 0.115 |
|  | Sleep hours | 0.127 | 0.086 | 1.478 | 0.140 |
|  | Low SMI | 0.600 | 0.330 | 1.820 | 0.069 |
|  | Power | 0.676 | 0.186 | 3.626 | 0.000 |
|  | Speed | 0.410 | 0.132 | 3.117 | 0.002 |
|  | Balance | 0.571 | 0.184 | 3.114 | 0.002 |
|  | Motor function | 0.677 | 0.573 | 1.182 | 0.237 |

Fit indices: CFI = 0.665, TLI = 0.279, RMSEA = 0.083, 90％ IC [0.000, 0.145]

The underlined paths from Low SMI to motor function were those considered unsuitable because their standardized path coefficients exceed 1.00.
